# Editorial: Biomolecular function and activity modulated by membranes

**DOI:** 10.3389/fmolb.2023.1133034

**Published:** 2023-01-24

**Authors:** Giray Enkavi, Jing Li, Hector Martinez-Seara

**Affiliations:** ^1^ Department of Physics, Faculty of Science, University of Helsinki, Helsinki, Uusimaa, Finland; ^2^ Department of BioMolecular Sciences, School of Pharmacy, The University of Mississippi, University, MS, United States; ^3^ Institute of Organic Chemistry and Biochemistry of the Czech Academy of Sciences, Prague, Czechia

**Keywords:** systems biology, atomistic molecular dynamics simultions, coarse-grained molecular dynamics simultions, membrane biology, lipids, membrane proteins, biomolecular function, biomolecular activity

Our knowledge of cellular membranes and their components’ critical role is recent and so far, unfinished. Bilayers of lipids were proposed less than 100 years ago (Gorter and Grendel, 1925). “Fluid mosaic model”, which explains fundamental issues like membrane organization, is just 50 years old (Singer and Nicolson, 1972). Many other core aspects are still a heated source of debate and discovery (Nicolson, 2014; Nieto-Garai et al., 2022). Today, we know that biological membranes are much more complex than any catch-all model, and almost every new investigation confirms this idea. In light of this, it is clear that more advanced experimental methods and more complex computational models, as well as finding ways to integrate the former two, are essential for the future of biological membrane research. This short Research Topic showcases four studies that use various methods to investigate membrane-related phenomena. Polasa et al. use an extensive set of equilibrium and non-equilibrium molecular dynamics simulations to describe the conformational changes in the YidC during membrane insertion (Polasa et al.). Similarly, Li et al. consider the effect of cholesterol and membrane composition on C99 dimerization with ramifications in Alzheimer’s disease (Li et al.). Be it atomistic or coarse-grained, molecular dynamics uses a physics-based approach to explicitly describe the interaction between molecular moieties of interest with an outstanding resolution. Aslam and Alvi present a different computational approach based on systems biology. Their mathematical modeling allowed the complete exploration of a novel postsynaptic channel for glutamatergic synaptic transmission and its effector molecules composed of ions, diacylglycerides, and protein kinases (Aslam and Alvi). Unlike molecular dynamics, systems biology works at much larger scales and aims to characterize emergent phenomena from complex and interdependent processes and interactions in biological systems. Advancing our knowledge of membrane-related processes depends on experimental methods as well. The Research Topic includes the purely experimental work by Kurakin et al., who studied lipid vesicle and divalent ion interactions (Kurakin et al.). This process is of paramount importance in signaling and membrane structure. Overall, we show that advancement in membrane research requires synergies from widespread techniques.
